# Advances in Nanoliposomes Production for Ferrous Sulfate Delivery

**DOI:** 10.3390/pharmaceutics12050445

**Published:** 2020-05-11

**Authors:** Sabrina Bochicchio, Annalisa Dalmoro, Gaetano Lamberti, Anna Angela Barba

**Affiliations:** 1 Eng4Life Srl, Spin-Off Accademico, Via Fiorentino, 32, 83100 Avellino, Italy; sbochicchio@unisa.it (S.B.); adalmoro@unisa.it (A.D.); glamberti@unisa.it (G.L.); 2 Dipartimento di Farmacia, Università degli Studi di Salerno, via Giovanni Paolo II, 132 84084 Fisciano (SA), Italy; 3 Dipartimento di Ingegneria Industriale, Università degli Studi di Salerno, via Giovanni Paolo II, 132 84084 Fisciano (SA), Italy

**Keywords:** nanoliposome, drug delivery, ferrous sulfate, simil-microfluidic apparatus, sonication

## Abstract

In this study, a continuous bench scale apparatus based on microfluidic fluid dynamic principles was used in the production of ferrous sulfate-nanoliposomes for pharmaceutical/nutraceutical applications, optimizing their formulation with respect to the products already present on the market. After an evaluation of its fluid dynamic nature, the simil-microfluidic (SMF) apparatus was first used to study the effects of the adopted process parameters on vesicles dimensional features by using ultrasonic energy to enhance liposomes homogenization. Subsequently, iron-nanoliposomes were produced at different weight ratios of ferrous sulfate to the total formulation components (0.06, 0.035, 0.02, and 0.01 *w*/*w*) achieving, by using the 0.01 *w*/*w*, vesicles of about 80 nm, with an encapsulation efficiency higher than 97%, an optimal short- and long-term stability, and an excellent bioavailability in Caco-2 cell line. Moreover, a comparison realized between the SMF method and two more conventional production techniques showed that by using the SMF setup the process time was drastically reduced, and the process yield increased, achieving a massive nanoliposomes production. Finally, duty-cycle sonication was detected to be a scalable technique for vesicles homogenization.

## 1. Introduction

One of the challenges of nanotechnology in the nutraceutical/food field is to improve the bioavailability of the nutritional components [1,2,3,4,5]. Indeed, many bioactive compounds are poorly absorbed and/or rapidly metabolized as soon as ingested and therefore are not able to be absorbed in their active form. Nanoscale delivery systems, such as nanoliposomes, can help to increase the bioavailability of active compounds, protecting them during the digestive process and, at the same time, improving their uptake in the gastro-intestinal tract [4,6,7,8]. Due to their unique properties of biocompatibility, biodegradability, low intrinsic toxicity and versatility, liposomes use has been rapidly grown as new technology for food fortification and dietary supplements (tablets/capsules) enrichment to increase active molecules shelf life [9,10,11].

Among the several applications of liposomal vectors already commercially exploited, the release of vitamins and micronutrients, as well iron in nutraceuticals, dietary supplements, and fortified foods constitutes the largest part [12].

Anemia is one of the most widespread nutritional deficiencies affecting globally two billion people, the prevalence of which, according to the World Health Organization, is due to the iron deficiency as well as dependent on ethnic groups and geographic location [13,14].

Several techniques have been used, until now, to produce systems for iron administration, and different are the corresponding commercial products available on the market (i.e., Sideral by Pharmanutra, Biofer by Lipotech, Ferosis Liposomal Drop by Viva Kids, F5 Fluidofer by AVD, Liposomal Iron by Yamamoto, Ferrum + C liposomal by ActiNovo). Although some of the current iron-based dosage forms have found their industrial application, the possibility to overcome operating disadvantages and to increase product quality is constantly being studied [15].

Today, the liposomal-iron based products found in the scientific literature and those already present on the market show some failing in terms of formulation. They are frequently characterized by an unbalanced ascorbic acid/iron ratio or contain the less absorbable iron form [9,16,17], i.e., the most used product against anemia, sold under the trade name *Sideral*, contains iron microencapsulated in a lipid matrix, but in this case, the iron is in the oxidation state +3 (Fe (III)). On the contrary, it is well known from the literature that the most bioavailable form of iron is Fe (II), presents in ferrous salts, such as ferrous sulfate [18,19]. It is also known that to further increase the iron bioavailability and promote its assimilation, it is necessary to assimilate it in association with a molecule capable of protection from oxidation, such as ascorbic acid or its salt, recommended in the ascorbic acid and iron weight ratio of 6:1 (*w*/*w*) by the World Health Organization (WHO) [20].

To increase product quality, the development of sustainable processes able to produce ad hoc liposomes in a rapid manner through the use of not labored techniques is the crucial point for the industrial sector. The techniques now used for the production of liposomal iron delivery systems (both as Fe (II) and Fe (III)), such as freeze-thawing (FT) [21], thin film hydration (TFH) [17], the ethanol injection method (EI) [22], and reverse phase evaporation (REV) [17], are all characterized by bulk discontinuous processes and low productivity [23,24,25]. The use of solvents that, although removed through evaporation stages, may constitute contaminants together with the use of inert atmospheres and other drastic conditions (such as low/high temperatures and pressures) make these techniques onerous even from an energetic point of view [26]. Moreover, products at nanoscale are not always obtainable by these methods, which are instead preferred compared to the micrometric scale for their larger interfacial surface area, which improves the solubility and enhances the bioavailability of the bioactive principle [27,28,29].

In the last few years, liposomes on a nanometric scale are being prepared, in a continuous manner, through microfluidics technology [30,31,32]. The method is characterized by elevated costs of microfabrication and low product volumes in output.

In order to produce higher volumes of iron-liposomal vectors directly on a nanometric scale and overcoming the previously mentioned operating limits, in this study, a robust, simple, and easy-to-transfer technology was realized. In particular, starting from the work of Pradhan et al. [33], a new continuous bench scale apparatus was designed and developed by exploiting microfluidics fluid dynamic principles transposed on a millimeter scale setup (carrying out a fluid dynamic similitude) and coupled with an ultrasound process used as intensification tool for vesicles homogenization. After the tuning of the process parameters in order to achieve the fluid dynamic similitude in the SMF setup according to the microfluidic principles, and the observation of their effect on vesicles dimension, nanoliposomes encapsulating ferrous sulfate were produced. First, the effect of different weight ratios of iron/total formulation components (0.06, 0.035, 0.02, and 0.01 weight ratio) on the final vesicles encapsulation efficiency was investigated.

Then, ferrous sulfate–loaded nanoliposomes with the best iron encapsulation efficiency, produced by the simil-microfluidic method, were chemical-physical characterized and subjected to short-term stability studies for six days at 4 °C and long term stability tests for six months at ongoing, intermediate, and accelerated conditions, according to the International Conference on Harmonization (ICH) guidelines [34].

Furthermore, bioavailability studies were performed by evaluating iron absorption after carrier uptake in CaCo2 cell line, used as a model of human gastrointestinal epithelial barrier [35].

A comparison in terms of vesicles final properties (number distribution size, calculated as the mean of the number size distribution, polydispersity index, zeta potential, charge, and encapsulation efficiency) and process yield was also realized between liposomes obtained using the simil-microfluidic (SMF) method and the ones obtained using the conventional techniques (ethanol injection (EI) and thin film hydration (TFH)), at the same operative conditions.

Finally, in order to perform propaedeutic studies for the plant scale up, the possibility to produce liposomal structures using sonication batch with higher volumes was tested.

## 2. Materials and Methods

### 2.1. Simil-Microfluidic Bench Scale Apparatus

#### 2.1.1. Fabrication

**Figure 1:**
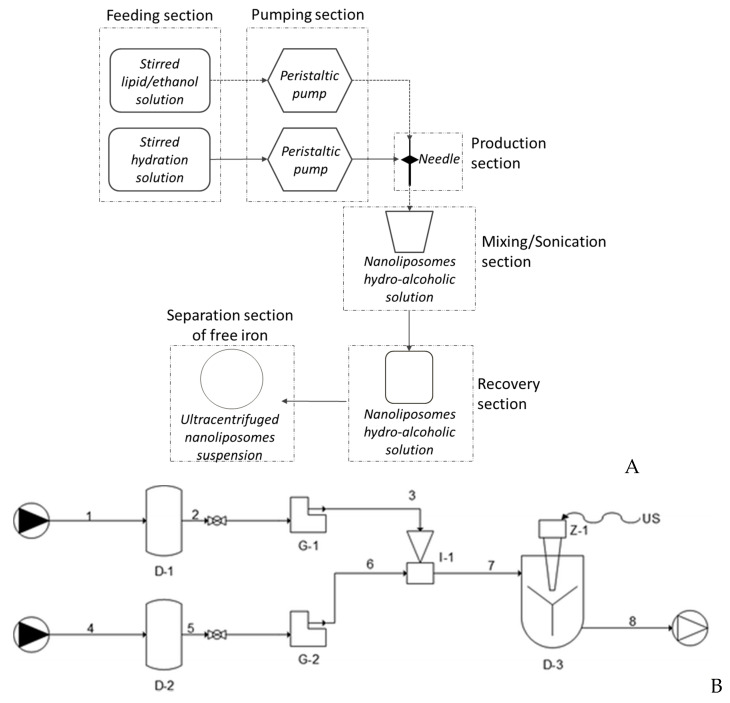
(**A**) Process schematization of nanoliposomes production through the simil-microfluidic apparatus; the main sections are reported: feeding, pumping, production, mixing/sonication, recovery, separation of free iron. (**B**) Piping representation for the experimental setup for the simil-microfluidic method: (1–2–3) lipids/ethanol feed line; (4–5–6) water feed line; (D-1 and D-2) feed tanks; (G-1 and G-2) peristaltic pumps; (I-1) injector (production section); (7–8) water/ethanol nanoliposomes suspension; (D-3) recovering/homogenizing tank (from Bochicchio, Dalmoro, Bertoncin, Lamberti, Moustafine and Barba [38] published by the Royal Society of Chemistry).

The fabricated simil-microfluidic apparatus is described in detail in Bochicchio Dalmoro, Recupido, Lamberti and Barba [36]. It is constituted by feeding, pumping, production, and recovery sections, as schematized in Figure 1 (A, process schematization, B, setup piping representation). In brief, two pipelines constitute the feeding sections; the first is composed of an agitated tank containing a lipids/ethanol solution, in which a hydrophobic active principle can be eventually dissolved, and the second is composed of a stirred tank filled with an aqueous phase, eventually containing an hydrophilic active ingredient. Two peristaltic pumps constitute the pumping section, while the production section is composed of the insertion of the lipids/ethanol solution pipe into a silicon tube, which is an extension of the water pipe. In this section, liposomes on a nanometric scale are formed as an effect of the interdiffusion phenomenon of the two pumped flows. Finally, separated from the rest of the plant, there is a homogenization section constitutes by an ultrasound device whose features are given in details in Bochicchio Dalmoro, Recupido, Lamberti and Barba [36]. After the scale up of the ultrasound assisted homogenization process, the plant configuration was slightly modified as detailed in Bochicchio, Lamberti and Barba [37] by including the homogenization section in the plant layout as described in Section 2.6.

To summarize the process, first, the lipids/ethanol and water solutions are pumped into the production section. Here, the hydroalcoholic suspension, containing liposomal vesicles, is stirred and then recovered in a container. Finally, in order to have homogenized vesicles, aliquots of the liposomal suspension are subjected to a duty cycle sonication process previously developed and modelled by Barba and collaborators, according to the process conditions reported in Barba, Bochiccho, Lamberti and Dalmoro [39].

#### 2.1.2. Flow Regime Evaluation

First, taking into account that a microfluidic system is characterized by a laminar flow regime, the fluid dynamic nature of the simil-microfluidic apparatus was evaluated on the bases of the Hagen-Poiseuille assumptions [40]. At first the Reynolds number (Equation (1), adopted to calculate the Re values reported in Table 1) was found for all the volumetric flow rates tested (10:1, 15:1, 20:1, and 40:1 hydration solution volumetric flow rate (Vhs) to lipid solution volumetric flow rate (Vls)). Afterward, in order to neglect the end effects, the “entrance length” was calculated and related to that of the piping in which the two phases interdiffuse. Parameters for calculations of the Reynolds number and entrance length are detailed in Bochicchio, Dalmoro, Bertoncin, Lamberti, Moustafine and Barba [38]. In particular, Reynolds number is defined by Equation (1):

(1) Re=4ρV˙πμD

where ρ, $\dot{V}$, and μ are density, volumetric flow rate, and dynamic viscosity of the fluid (ethanol–organic phase; water–polar phase; fluid approximation for hydroalcoholic suspension–water), respectively; π and D are the mathematical constant 3.14 and the internal tube diameter, respectively.

#### 2.1.3. Process Parameters Optimization

Once the geometric parameters are defined, those of the process such as the volumetric flow rates, the lipid concentration, and the sonication conditions adopted were investigated in order to establish their control on the nanoliposomes dimensional features as in depth described Bochicchio Dalmoro, Recupido, Lamberti and Barba [36]. Shortly, unloaded vesicles were at first prepared by maintaining constant the PC concentration in the hydroalcoholic solution at 0.15 mg/mL and varying the volumetric flow rates (10:1, 15:1, 20:1, and 40:1 Vhs/Vls). Subsequently, maintaining the Vhs/Vls ratio at 10:1, the lipid concentration in the final hydroalcoholic solution was changed (0.15, 1, 4, and 5 mg/mL).

For all the process conditions tested, liposomes size, expressed as the mean of the number size distribution and called “number distribution size”, and PDI were analyzed with and without ultrasonic energy contribution as described in Section 2.3.2.

### 2.2. Ferrous Sulfate Loaded Nanoliposomes Production

#### 2.2.1. Materials

L-α-Phosphatidylcholine (PC) from soybean, Type II-S, 14–23% choline basis (CAS n. 8002-43-5), Cholesterol (CHO) (CAS n. 57-88-5), Rhodamine B dye (CAS 83-68-9), ferrous sulfate heptaidrate (CAS n. 7782-63-0), ascorbic acid (CAS n. 50-81-7), ethanol of analytical grade (CAS n. 64-17-5), Triton X100 (CAS n. 9002-93-4), hydrochloric acid (CAS n. 7647-01-0), hydroxylamine hydrochloride (CAS n. 5470-11-1), ammonium acetate (CAS n. 631-61-8), glacial acetic acid (CAS n. 64-19-7), and 1,10-Phenantroline (CAS n. 66-71-7) were purchased from Sigma Aldrich (Milan, Italy).

#### 2.2.2. Nanoliposomes Preparation through the Simil-Microfluidic Apparatus

Ferrous sulfate loaded nanoliposomes were prepared adopting the optimized conditions of 10:1 Vhs/Vls and 5 mg/mL lipids in the final hydro alcoholic suspension. To this aim, in 10 mL of ethanol were dissolved 94 mg of CHOL and 470 mg of PC. CHOL was added to the formulation in order to stabilize the loaded vesicles and in particular the ratio 2.5:1 (mol/mol) PC/CHOL CHOL was used. This ratio corresponds to the typical composition of the cell membrane, as suggested by Abbasi and Azari [41]. Ferrous sulfate heptaidrate and ascorbic acid were carefully dissolved in 100 mL of deionized water. This solution was then used as a hydration solution. Ascorbic acid, in a ferrous/ascorbic acid weight ratio of 1:6 (*w*/*w*), was added to preserve the ferrous ion against oxidation phenomena. Four different nanoliposomal lots were produced. Starting from a 0.06 ferrous sulfate/total components weight ratio, indicated in Xia and Xu [17], the four different nanoliposomal formulations were prepared by using a 0.035, 0.02, and 0.01 ferrous sulfate/total components (*w*/*w*) ratio. For each kind of formulation, an unloaded sample was produced (and used as control) adopting the same manufacturing conditions and using only deionized water as hydrating phase. All the lots were produced in a homogenized and not homogenized form by treating 1 mL sample aliquots with a 3 mm sonotrode, by applying a 45% amplitude duty cycle consisting of five 10-seconds irradiation rounds each, followed by a 20 s pause (130 W, 20 kHz) [39].

#### 2.2.3. Nanoliposomes Preparation through Two Classical Bench Scale Techniques

The conventional ethanol injection (EI) and the thin film hydration (TFH) methods were adopted for the production of ferrous sulfate loaded nanoliposomes in order to have a comparison in terms of productivity with those achieved by the use of the SMF apparatus. In particular, the fourth formulation with a 0.01 ferrous sulfate/total components (*w*/*w*) ratio was produced with both the techniques, and the liposomes number for unit volume of solution (*N_lip_*) obtained was analytically calculated, as done by *Encapsula Nanosciences*, a liposome formulator and manufacturer pharmaceutical company, considering the following equation:

(2) $$N_{lip}=\frac{M_{lip}\cdot N_{a}}{N_{T}\cdot 1000}$$

where *ML_ip_* is the PC concentration (mol/mL); *N_α_* is the Avogadro number (6.022·10^23^/mol); $N_{T}$ (nm^2^/nm^2^) is the lipids number presents in one liposome depending on liposomes diameter and determined by using the relation

(3) $$N_{T}=\frac{[4\cdot \pi \cdot \left(\frac{d}{2}{)}^{2}+4\cdot \pi \cdot {\left(\frac{d}{2}-h\right)}^{2}\right]}{a}$$

where *d* is the vesicles diameter expressed as the number distribution size (nm), *h* is the thickness of the bilayer (nm) and *a* is the hydrophilic portion surface area (nm^2^).

For unilamellar vesicles made from phosphatidylcholine, the bilayer thickness can be considered of about 5 nm while the hydrophilic portion surface equivalent to 0.71 nm^2^ [42].

##### Ethanol Injection

Ferrous sulfate–loaded nanoliposomes were prepared by dissolving 470 mg of PC and 94 mg of cholesterol in 10 mL of ethanol. After stirring, the ethanol/lipid solution was sprayed by means of a 10 mL syringe (needle of 0.8 mm) in a 100 mL of deionized water containing ferrous sulfate (5.64 mg) and ascorbic acid (33.8 mg) at 1:6 (*w*/*w*). Liposomes suspension was stirred for 10 min and left stabilize at room temperature for two hours. Finally, aliquots (1 mL) of the obtained vesicles were subjected to the duty cycle sonication homogenization process, according to the procedures previously described [38]. In this way, nanoliposomes were produced by maintaining the lipid concentration in the final hydroalcoholic solution at 5 mg/mL and the volume ratio between the hydration solution to the lipid solution at 10:1 (*v*/*v*) using the simil-microfluidic apparatus in optimized conditions.

##### Thin Film Hydration

The TFH method was also used to produce nanoliposomes maintaining a constant lipid concentration and PC/CHOL ratio. Briefly, 47 mg of PC and 9.4 mg of CHOL at 2.5:1 (mol/mol) ratio were dissolved in 1 mL of chloroform/methanol at 2:1 (vol/vol). The solvent was removed by evaporation at 50 °C for 3 h in a rotary evaporator (Heidolph, Laborota 4002 Control, Bergamo, Italy) under reduced pressure until a lipid film was formed. The dried lipid film was then hydrated at room temperature with 11 mL of deionized water containing ferrous sulfate (0.62 mg) and ascorbic acid (3.72 mg) at 1:6 (*w*/*w*). The preparation containing multilamellar vesicles (MLVs) was maintained at room temperature for 2 h, and subsequently, sample aliquots (1 mL) were subjected to the ultrasound assisted homogenization process, in the mode previously described [39].

### 2.3. Vesicles Characterization

#### 2.3.1. Morphology

Morphological characterizations of unloaded and ferrous sulfate–loaded nanoliposomes were performed by transmission electron microscopy (TEM) (EM 208, Philips, Amsterdam, Netherlands) equipped with camera Quemesa (Olympus Soft Imaging Solutions, Münster, Germany). The samples were negatively stained with 1% (*w*/*v*) of uranyl acetate solution.

Fe-nanoliposomes were also observed by the optical microscope in fluorescence field Axioplan 2- Image Zeiss, Jena, Germany, equipped with software to capture the images. The Rhodamine B dye was used to visualize lipid vesicles with a 100 X oil immersion objective.

#### 2.3.2. Size and Zeta Potential

Size and zeta potential determinations of unloaded and ferrous sulfate loaded vesicles were performed by Dynamic Light Scattering using the Zetasizer Nano ZS (Malvern, UK) with noninvasive backscatter (NIBS) optics. In particular, 100 µL aliquot of the sample and 900 µL of deionized water (pH 6, measurements at room temperature) were transferred into the appropriate reading cuvettes and a detection angle of 173 degrees was used. Particles size was expressed as the intensity based harmonic mean (the Z-Average) and as the mean of the number size distribution called “number distribution size”, made up by plotting the number of particles versus the particle size. Polydispersity Index (PDI), a parameter that indicates the distribution of size populations within a given sample [28] (0—perfectly uniform sample with respect to the particle size; 1—highly polydisperse sample with multiple particle size populations) was measured for all the preparations.

All the measurements were performed in triplicate; the results were expressed as average values. Statistical analysis of experimental data (size and PDI) were analyzed using Student’s t-test and one-way analysis of variance (ANOVA); differences were considered statistically significant at *p* < 0.05.

#### 2.3.3. Encapsulation Efficiency (E.E.)

From all the samples produced, aliquots were taken and spectrophotometrically analyzed in order to measure the real encapsulated and the un-encapsulated amount of ferrous sulfate. For this purpose, 2 mL aliquots of the sample were centrifuged at 30,000 rpm (87,019× *g*) (Beckman Optima L-90K, SW 55 Ti rotor) for 1 h at 4 °C in order to remove the supernatant from the precipitated nanoliposomes (pellet). The supernatant volume, stored for the subsequent ferrous sulfate determination, was measured and replaced with the same volume of Triton X100 at 1% (*v*/*v*) in order to lyse the nanoliposomes pellet and to analyze the encapsulated ferrous sulfate.

Iron determination was performed by the 1,10-Phenanthroline assay. Briefly, to 400 μL aliquot of supernatant, 200 µL of 37% (*v*/*v*) hydrochloric acid were added to acidify the solution together with 100 μL of 10% (*v*/*v*) hydroxylamine hydrochloride, used as a reducing agent. After 10 min, 1 mL of ammonium acetate buffer solution, previously prepared by dissolving 50 g of ammonium acetate in 30 mL of deionized water and 140 mL of glacial acetic acid, was added together with 200 μL of 1,10-Phenantroline at 0.5% (*v*/*v*). The steps were repeated for the pellet dissolved in Triton X100. The total volume for each test tube was then increased to 10 mL with deionized water. Blanks reagents were prepared in the same manner but using pure water or Triton X100 in place of the samples. An absorption spectrum of 200–600 nm was investigated for all the samples and the maximal wavelength of 510 nm, typical of the 1,10-Phenantroline-Fe^2+^ ions complex, was considered. The iron amounts were assayed by using a ferrous sulfate calibration curve previously prepared with the same 1,10-Phenantroline colorimetric determination. The UV spectrophotometric analysis was performed using a Lambda 25 UV/VIS Spectrophotometer (PerkinElmer, Monza, Italy).

The encapsulation efficiency (E.E.) was determined as the percentage of ferrous sulfate encapsulated in nanoliposomes to the initial amount of ferrous sulfate included in the formulation and was calculated using Equation (4):

(4) $$E.E.\left(\%\right)=\left(\frac{Fe\;in\;the\;pellet,\;\;mg}{Fe\;in\;the\;pellet+Fe\;in\;the\;supernatant,\;mg}\right)\times 100$$

Iron loads were determined through the ratio:

(5) $$\mathrm{Load},\%=\frac{Fe,\;\;mg}{Fe+ascorbic\;acid+lipids,\;mg}\times 100$$

In particular, the theoretical load refers to the initial amount of iron included in the formulation divided by the total mass, while the effective load refers to the iron amount effectively presents in the nanoliposomes (pellet) divided by the total mass.

### 2.4. Stability Test

The nanoliposomes produced by the simil-microfluidic method, with the best iron encapsulation efficiency (fourth formulation), were tested for short- and long-term stability, as described below.

#### 2.4.1. Short-Term Stability

The stability of nanoliposomes containing ferrous sulfate were tested in conditions simulating those of food/beverage storage. In particular, the samples were maintained at 4 °C for a period of about six days in deionized water (pH 6), and the mass of iron present in the supernatant and in the pellet was quantified at established times. Briefly, given amounts of loaded nanoliposomes were put in measured volumes of deionized water. Subsequently, after 6 h, 22 h, 46 h, 70 h, and 130 h, sample aliquots were taken, centrifuged, and treated under the same operating conditions described for the encapsulation efficiency determination. All the measurements were performed in triplicate.

#### 2.4.2. Long-Term Stability

Ongoing, intermediate, and accelerated stability were tested as recommended by the International Conference on Harmonization (ICH) guidelines [34]. In particular, three different batches of ferrous sulfate loaded nanoliposomes were produced, without the use of any stabilizing additives, and stored for six months at environmental conditions 25 °C ± 2 °C/60% RH ± 5% RH (ongoing stability, e.g., stability observed at room conditions at real time); at 30 °C ± 2 °C/65% RH ± 5% RH (intermediate stability, e.g., stability observed at stressed conditions); and at 40 °C ± 2 °C/75% RH ± 5% (accelerated stability, e.g., stability observed at more stressed conditions) under thermostat conditions, in the absence of light. Samples were taken at specific time points (0, 1, 3, and 6 months); observed for their visual aspect by taking photographs; and tested for particles number distribution size, PDI, Z-average, zeta-potential, ferrous sulfate encapsulation efficiency (using the same operating conditions described in Section 2.3), and sample volume variation, by weighing the samples after 1, 3, and 6 months and noting the difference with the sample weight at zero time.

All the measurements were performed in triplicate. Statistical analysis of experimental data was analyzed using Student’s t-test, and differences were considered statistically significant at *p* < 0.05. The samples’ ongoing stability is still under evaluation.

### 2.5. In Vitro Bioavailability Studies

In order to test the ability of liposomal formulations to overcome gastrointestinal epithelial barriers, the CaCo2 cell line, successfully used in previous works for the evaluation of iron absorption [43,44,45,46], has been proposed. Here, 10^4^ Caco-2 cells were seeded in 25 cm^2^ culture flasks. Cells were cultured in Dulbecco’s modified Eagle’s high glucose medium supplemented with 10% heat-inactivated fetal bovine serum, 100 U/mL penicillin, 100 µg/mL streptomycin and 2mM L-glutamine (EuroClone, Italy) at 37 °C in a humidified atmosphere containing 5% CO_2_.

Fourteen days post seeding, when the cultures reached 90% confluence, the cells were treated for 2 h with a liposomal iron concentration of 20 µM. After 24 h, cells and medium from cultures (supernatant) were collected separately. Total protein extracts were obtained after lysis of pellet cells in RIPA Buffer (Sigma-Aldrich, St Louis, MO, USA) supplemented with protease and phosphatase inhibitor cocktails (Sigma-Aldrich, USA). Total protein concentration was measured by BCA protein assay reagent (Pierce, Rockford, IL, USA).

Ferritin content in the protein lysates was evaluate by the automated “Access Ferritin” immunoassay (Beckman Coulter). The concentration of extracellular ferritin released from the cells in the culture medium at 24 h after treatment with nanoliposomes was also evaluated by measuring the ferritin concentration in the cell supernatants.

Ferritin concentration, used as a marker of iron absorption, was reported on total protein concentration (ng ferritin/mg protein).

### 2.6. Scale-Up of the Ultrasound Assisted Homogenization Process

To move from a production scale of a few milliliters to a greater production batches (of the order of hundred milliliters) the set-up described in 2.1.1 paragraph has been modified. In particular, the 3 mm sonotrode tip was replaced by a 6 mm tip acting directly on the entire volume of the liposomal suspension (110 mL per batch). In order to obtain, with the new plant configuration, the same final nanoliposomes dimensional features previously achieved by sonicating 1 mL aliquots of sample, the duration of each cycle (10 s) was kept constant and the time in which the ultrasound is not applied (20 s), and operating with an amplitude of 100%, the ultrasound power and the number of cycles required to sonicate a 110 mL volume of liposomal suspension were determined. In particular, starting from a volume of unloaded nanoliposomes suspension of 1 mL, using an amplitude of 100%, the energy supply for one sonication cycle of 10 s was determined. Considering the linearity between the power and the volume of sonication, the power required to sonicate 110 mL of liposomal suspension was determined, and then the number of duty cycle sonication rounds able to provide the necessary power were obtained experimentally. In particular, unloaded nanoliposomes were produced by maintaining constant the optimized conditions (10:1 Vhs/Vls; 5 mg/mL lipids/final hydro alcoholic1 solution) and by submitting the samples to 15, 30, and 60 duty cycle rounds. Finally, vesicles were characterized in terms of number distribution size and PDI.

## 3. Results and Discussion

### 3.1. Simil-Microfluidic Apparatus

In this work, a novel bench scale apparatus for ferrous sulfate-nanoliposomes production was successfully designed and realized, overcoming the limits imposed by the conventionally used techniques. In particular, by coupling the ethanol injection method with the microfluidic fluid dynamic principles and transposing them on a millimetric scale it was possible to achieve a massive nanoliposomes output in an easy and cheap way. Indeed, in a work of Pradhan and collaborators [33] a syringe pump driven microfluidic device was developed to produce nanoliposomes of controlled dimensions but achieving small product volumes in output. Here the continuous bench scale apparatus was designed by replacing the syringe pumps by the peristaltic one, allowing the production of larger volumes and by injecting the lipid phase directly into the polar phase without the help of any tubes connection systems.

It should be stressed that small output volumes of supplemental products (usually ranging from 10 to 60 mL) are directly linked with their high commercial costs which represent the reason why they are not jet widely used as a very proper therapy. With the simil-microfluidic setup developed it was possible to obtain a massive output with the minimum of energy, costs and time: one batch of 110 mL iron-liposomal suspension can be produced in few minutes. Moreover, microfluidics-based methods, such as the MHF platform made by Jahn et al. [47], which are expensive due to special devices needed and microfabrication costs, have been transposed to a millimeter scale, drastically reducing the production costs and increasing the yields.

The simil-microfluidic technique overcame the limit of bulk methods in which it is difficult to control liposomes dimensional features; instead, they were carefully modulated in this work through a further ultrasound-assisted homogenization step. Finally, the setup developed allowed us to produce ferrous-sulfate nanostructures without the use of drastic conditions, such solvents and/or high pressure, currently used in literature and also at industrial scale, i.e., Reverse Phase Evaporation, Thin Film Hydration, and Homogenization-Freeze Thawing methods; the last method is used for the production of the commercial iron *Biofer^®^* (Lipotech, European reference: Barcelona, Spain) [9,17,21,41].

#### 3.1.1. Fluid Dynamic and Phenomenological Aspects

Like in a microfluidic system, due to the millimeters scale of the channels and the low volumetric flow rates, in the developed simil-microfluidic apparatus the flow is laminar in opposition to the chaotic condition characterizing the bulk phases. Indeed, the results relative to the flow regime evaluation have shown that all the Hagen–Poiseuille assumptions were satisfied [39].

**Figure 2:**
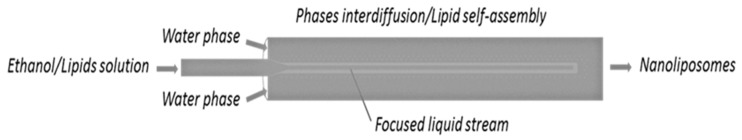
Representation of the liposome formation as an effect of the interdiffusion phenomenon occurring during vesicles production through the microfluidic approach.

As shown in Table 1, for all the conditions tested, the polar and the organic phases exhibit a laminar flow regime in the feeding section. Likewise, the hydroalcholic phase in the production section. In particular, the Reynolds number changes from 311.4 to 1160, moving from 10:1 Vhs/Vls to 40:1 Vhs/Vls. This result shows that the blending between the two phases is governed by diffusion mechanisms. Apart from the Reynolds number, the other Hagen–Poiseuille assumptions were also satisfied. In particular, the piping length in which the two phases interdiffuse is longer than the “entrance length” needed to build up the parabolic profile—33 mm and 122 mm entrance lengths were found at the lower and the higher Reynolds number tested, respectively. These lengths are lower than that of the pipe (150 mm) where interdiffusion phenomena occurred. As described in references [40,48], from a phenomenological point of view, liposomes are formed as an effect of the molecular diffusion between the water and the lipid/ethanol phases, which starts at their interface (Figure 2). During this process, lipid vesicles start to “self-assemble” in the form of bilayer fragments (phospholipid bilayer fragments, BPFs) [49], whose solubility in the solvent is slowly reduced by the presence of water (which is spreading). This thermodynamic instability causes the curvature of the BPFs and their closure in a spherical configuration, thus allowing the formation of liposomal vesicles [49]. The modulation of the insertion section of the organic phase into the aqueous phase can result in a change of vesicles dimension. In particular, it was shown by Jahn et al. that what plays a key role is the volumetric flow rate ratio between the two liquids [50], as also confirmed in this work and described in the next subsection.

#### 3.1.2. Process Parameters Optimization

The volumetric flow rate ratio exerts a strong influence on the final vesicles dimensional features as also found by Jahn et al. for a microfluidic hydrodynamic focusing platform [47] and as described in our previous work [36,40,48].

**Figure 3:**
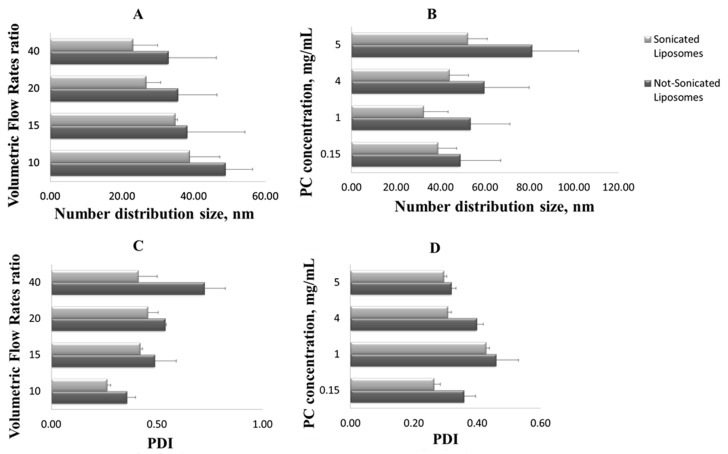
Sonicated and not sonicated liposomes number distribution size (the mean of the number size distribution) at different volumetric flow rate ratios (**A**) and L-α-Phosphatidylcholine (PC) concentrations (**B**). Sonicated and not sonicated liposomes polydispersity index (PDI) at different volumetric flow rate ratios (**C**) and PC concentrations (**D**). Results are expressed as average of three determinations and reported along with the standard deviation.

Specifically, referring to the number distribution size, not sonicated liposomes of 49 nm were obtained by operating with a 10:1 Vhs/Vls ratio, and even smaller vesicles were achieved with a 15:1 and 20:1 Vhs/Vls, up to a diameter of 33 nm for liposomes obtained using a 40:1 Vhs/Vls, which was the higher volumetric flow rate ratio tested (Figure 3A). When the lipids concentration increases, the liposomes size rises—at 0.15 mg/mL concentration, vesicles of 49 nm in size are formed, and at 5 mg/mL, liposomes with a diameter of 81 nm are found (Figure 3B). In particular, the reduction of the liposomes size is an effect of the diffusion rate of the lipids into the hydration solution—as the hydration solution volumetric flow rate increases, lipids, impacting on a larger volume and being distant from each other, aggregate to form smaller vesicles. On the contrary, as the lipid concentration increases, phospholipids, impacting at the same alcohol/water interface area, are closer to each other and thus form larger vesicles [36,37].

Finally, the sonication process has confirmed to be efficacy for liposomes homogenization ameliorating liposomes dimensional distribution and reducing the polydispersity of the sample (values statistically significant, *p* < 0.05); i.e., for the 40:1 Vhs/Vls, the PDI is nearly halved (Figure 3C), and for the 5 mg/mL concentration, after the homogenization step, the PDI decreases from 0.32 to 0.29 (Figure 3D).

Summarizing, the results relative to the unloaded vesicles production have shown that liposomes with the best size distribution were produced by using a volumetric flow rate ratio of 10:1 Vhs/Vls and a concentration of lipids equal to 5 mg/mL.

### 3.2. Ferrous Sulfate Loaded Nanoliposomes Production through the Simil-Microfluidic Apparatus

#### 3.2.1. Loaded Liposomes Formulation

Scientific works about iron encapsulation in lipid particles for nutraceutical applications show some gaps in terms of formulation efficacy selecting the less absorbable form of iron, the ferric form [16] and an unbalanced ascorbic acid/iron (AA/Fe) weight ratio compared to that recommended by the World Health Organization (WHO) [18]. It has been shown that the co-addition of ascorbic acid and iron in a 2:1 molar ratio (6:1 weight ratio) increases iron absorption from foods two- to three-fold in adults and children [51,52,53]. Nevertheless, a 1:15 weight ratio of ascorbic acid to ferrous sulfate was used by Xia and Xu [17] and also by Abbasi and Azari [41] while a 1:1.5 AA/Fe weight ratio was used by Kosaraju and collaborators [9]. Even if referring to the products already present on the market, we can find that *SunActive^®^*, a TAIYO International (European reference: Schwelm, Germany) product for iron fortification, contains a 4:1 *w*/*w* A.A./Fe, furthermore the ferric pyrophosphate is used in this formulation. Biofer^®^, a Lipotech (European reference: Barcelona, Spain) microencapsulated iron product, contains 1:15 AA/Fe *w*/*w*. Sideral and Sideral Forte, a Pharmanutra products, are formulated with the less absorbable ferric pyrophosphate form and contains from 1:4 to 1:2 *w*/*w* Fe/AA. This last ratio is used in Sideral Forte for the treatment of severe anemias. In this work, the formulation was optimized using the most absorbable form of iron, ferrous sulfate, together with the correct 1:6 Fe/AA weight ratio.

#### 3.2.2. Vesicles Characterization

##### Morphology

**Figure 4:**
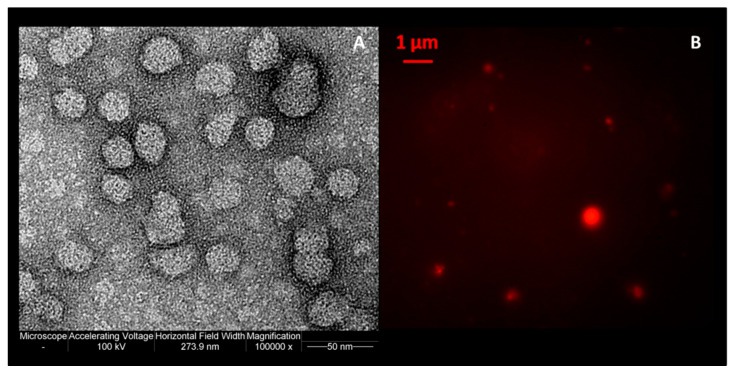
(**A**) Transmission electron micrograph; (**B**) fluorescence microscopy images of ferrous sulfate lipid vesicles prepared by using the simil-microfluidic method.

In order to obtain information about morphology, transmission electron microscopy (TEM) (Figure 4A) and fluorescence imaging (Figure 4B) were used. As showed in Figure 4, spherical and separated liposomes were achieved with all the formulations tested.

##### Size and Zeta Potential

Generally, the iron solubility is very dependent on the size and the shape of the iron-particles complexes, characteristics which are governed by the manufacturing process [54]. In that regard, particles of nanometric scale are required to maintain the transparency of clear beverages during enrichment—carrier have to be small enough so as not to scatter light and be detected by the naked eye [55]. The nanoscale plays a crucial role for other forms of supplementation, such oral formulations. In this case, the size of delivery systems has a remarkable influence on the carrier’s uptake after their administration; in many works, it has been proved that nanostructured delivery systems yield an increase in drug uptake, enhancing the intestinal absorption of the active principle [56,57].

In this work, ferrous sulfate-liposomes of nanometric size were produced (Table 2). In particular, in reference to the samples that were not sonicated, it can be observed that the diameter size, expressed as number distribution size, of unloaded nanoliposomes (about 72 nm) is smaller than that of the loaded form. The increases of loaded vesicles average dimension, of 103–154 nm range size, is indicative of the fact that a certain amount of iron and ascorbic acid is encapsulated within the vesicles but also of the fact that a possible particles aggregation may have occurred. Considering the homogenized samples, it can be note that the sonication has a crucial role in reducing the PDI of the loaded vesicles, confirming what was previously seen for the unloaded structures, i.e., the average liposomes PDI of the 0.06 *w*/*w* formulation initially of 0.45 ± 0.04, has become 0.38 ± 0.01 after the ultrasound-assisted step. A reduction in PDI by passing from the not sonicated to the sonicated liposomes was always found for all the samples tested (Table 2).

Moreover, it is important to note that the number distribution size and PDI values obtained for the unloaded liposomes not sonicated with the addition of cholesterol (72 nm; 0.4 PDI) are near to the value obtained for the unloaded nanoliposomes not sonicated formulated with only PC (81 nm size; 0.32 PDI, Figure 3B) using the same process conditions of 10:1 Vhs/Vls and 5 mg/mL lipids concentration. The comparison points out the reliability and the reproducibility of the developed set-up and is also true for the sonicated samples. However, it should be noted that, despite the effectiveness of the homogenization process adopted and the previous efforts made to make the US process the least invasive and as short as possible [38], a possible degradation of phospholipids during the sonication procedure, the eventual pollution of the sample by metal residues coming from the tip of the sonicator, and the formation of larger vesicles or aggregates in the sample could occur. This could lead to relatively high PDI values (Table 2).

Another important parameter to take into account in the development of drug delivery systems to be used in nutraceutical applications is the zeta potential (ζ), a stability index of the nanoliposomes suspensions. In order to avoid the nanostructures aggregation, resulting in a precipitation and loss of product stability, carriers should have a |ζ| > 30 mV [58]. Here, negatively charged vesicles were produced for all the formulations tested (0.06, 0.035, 0.02 and 0.01 *w*/*w*). In particular, ζ for unloaded liposomes was negative (−57.87 ± 1.13 mV) due the presence of polyunsaturated fatty acids (linoleic and oleic acids) composing the phosphatidylcholine vesicles and due to the fact that the hydroxyl groups of the cholesterol polar head are easily combined with choline in the polar region of PC, producing a kind of dipole tropism that increases the liposomal surface negative charges [17,59]. Instead, as a consequence of the presence of positive-charged iron ions, the vesicles surface becomes less negative for loaded liposomes (Table 2). In particular, it seems that for the 0.035 and 0.02 (*w*/*w*) formulations the electrostatic interactions between iron and lipids have concerned more the outer vesicles surface than the core, being the zeta potential less negative (about −20 mV) respect to the other formulations (about −41 mV and −36 mV) where iron is mostly entrapped in the liposomes core (see Table 2).

##### Encapsulation Efficiency (E.E.)

In order to make iron unreactive during its administration and storage, ferrous sulfate was isolated from the external environment and oxidizing agents by encapsulation in liposomal structures. In particular, according to Xia and Xu, it was observed that increasing ferrous ion concentration there is a decreases of encapsulated ferrous ion amount when deionized water is used as hydrating medium [17]. Nevertheless, it is important to underline that by using the Reverse-Phase Evaporation (REV) method and maintaining the same hydration medium and iron species used in this work, Xia and Xu have obtained an encapsulation efficiency (E.E.) of about 5% and 16%, respectively, for a 0.06 *w*/*w* and a 0.04 *w*/*w* iron/total components used. As visible from Table 3, in this work, a 22% E.E. and a 42% E.E. were achieved by using respectively the same iron/total formulation components with the use of the simil-microfluidic technique. In general, the decreases in iron E.E. by increasing the ferrous sulfate concentration can be explained by the fact that the ferrous sulfate is a strong electrolyte, and for major load values, the ionic strength, defined as the concentration of ions present in a solution, increases, resulting in a reduction of the encapsulation capacity in the aqueous phase (solution of iron and ascorbic acid) [55]. In virtue of these considerations, loaded nanoliposomes characterized by higher iron/total formulation components weight ratios were studied observing an increased E.E., in particular of 52% by using a 0.02 *w*/*w* iron/total components and of about the 97% with the 0.01 *w*/*w* iron/total components formulation (Table 3).

In general, better E.E. results have been obtained by the use of a simple and rapid technique and just deionized water as hydration medium. Indeed, by means of the last formulation, it was possible to obtain stable liposomes with a diameter of 76 nm and an encapsulation efficiency of 97%. In a work by Kosaraju et al., a preparation of Fe/AA-loaded liposomes below 250 nm in size was obtained for Fe/AA concentrations of 7.5/5% (*w*/*w*) using *Emultop^®^* and obtaining a maximum of 57% E.E. with an homogenization process [9]. In some cases, higher ferrous sulfate E.E. were obtained in liposomes by using citric acid sodium phosphate buffer solution as hydration medium and Tween 80 in the lipid formulation but the liposome dimensions were in the micrometer scale [41].

#### 3.2.3. Stability Test

Short-Term Stability

**Figure 5:**
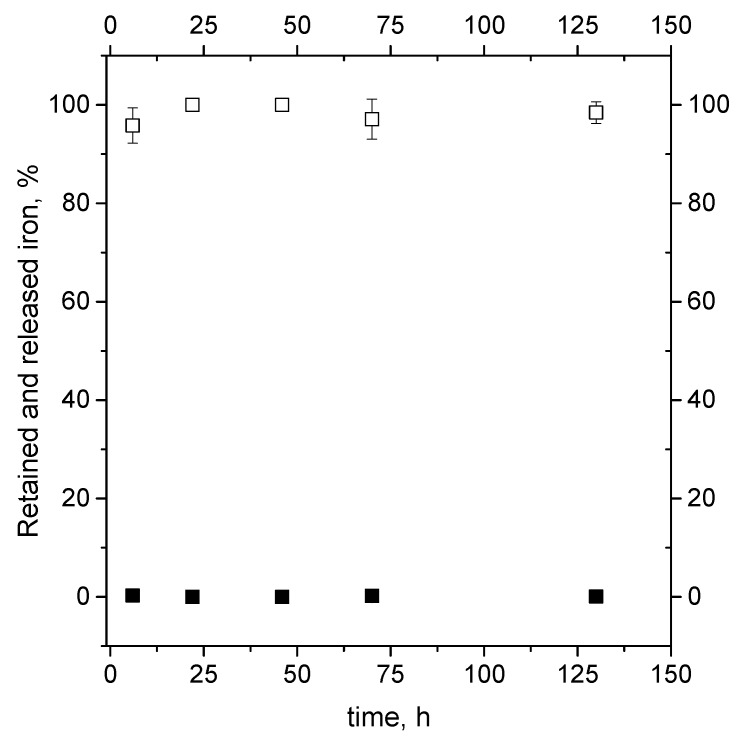
Assayed percentage of iron retained in nanoliposomes (open symbols) and released in deionized water (closed symbols) during short-term stability test of samples stored in water at 4 °C for six days.

The stability of the produced iron loaded nanoliposomes with the best encapsulation efficiency (0.01 *w*/*w* Fe/total formulation components) was tested in conditions simulating those of iron enriched food (such milk or fruit juice) storage. The samples, maintained at 4 °C for a period of about six days in deionized water, were found to be stable, as can be observed from Figure 5.

Indeed, the trend of the mass of iron presents in the pellet and in the supernatant at different time remains constant during the observation period, showing the liposomes to have a good ability to retain the iron mass in their core structures and thus proving to be useable for food iron enrichment application.

##### Long-Term Stability

The assurance of safety, quality, and efficacy of a drug product largely depends on its stability during its lifecycle, and this is the reason why the industries are now standardizing stability testing protocols on the recommendations of the International Conference on Harmonization (ICH) [60].

On the basis of a possible industrialization of the formulation produced, in this work, the stability of the ferrous sulfate-loaded nanoliposomes was tested according to ICH guidelines. The trends of particles number distribution size, Z-average, PDI, Zeta-potential, ferrous sulfate encapsulation efficiency, and sample volume variation at 25 °C ± 2 °C/60% RH ± 5% RH (ongoing stability), 30 °C ± 2 °C/65% RH ± 5% RH (intermediate stability), and 40 °C ± 2 °C/75% RH ± 5% (accelerated stability) storage conditions was investigated over six months. A visual inspection of the samples to check the presence of agglomerations was also carried out.

**Figure 6:**
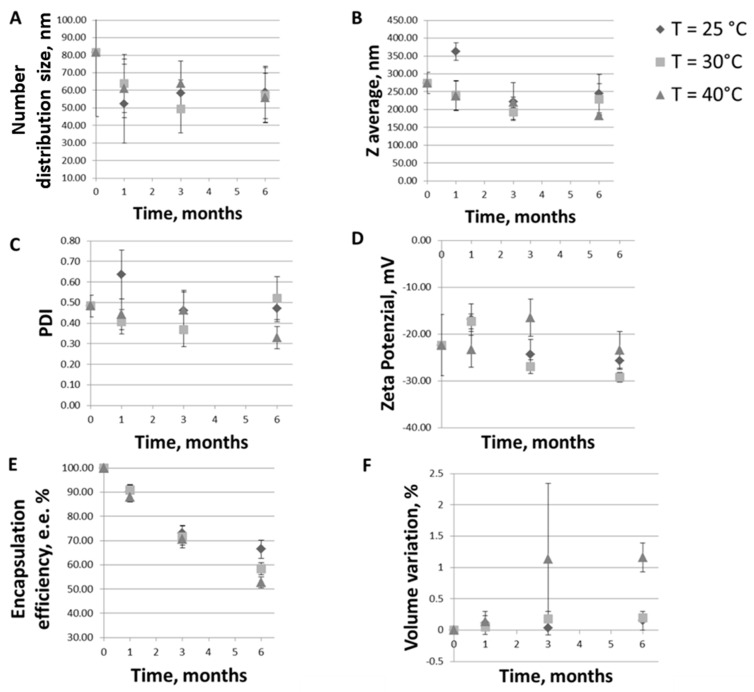
Trends of (**A**) particles number distribution size (the mean of the number size distribution); (**B**) Z-average; (**C**) PDI, (**D**) Zeta-potential, (**E**); ferrous sulfate encapsulation efficiency; (**F**) sample volume variation during six months sample storage at 25 °C ± 2 °C/60% RH ± 5% RH (ongoing stability, rhombus symbols), 30 °C ± 2 °C/65% RH ± 5% RH (intermediate stability, square symbols) and 40 °C ± 2 °C/75% RH ± 5% (accelerated stability, triangle symbols) conditions.

Fusion and aggregation can be affected by the lipid components of the liposome [61], and, as a consequence of fusion, vesicles are susceptible to an increase in particle size. Therefore, monitoring the evolution of liposome dimension during storage is fundamental. As shown in Figure 6A, the number distribution size remained in the same range of values (50–80 nm) for up to six months at all the temperatures tested (slight differences were not significant, *p* > 0.05). The nanoliposomes stability was also confirmed by Z-average (Figure 6B), PDI (Figure 6C), and Zeta-potential (Figure 6D) values that, except for a rapid increase after one month for the ongoing stability tested sample (probably due to the swelling behavior of charged phospholipids due to ion hydration [62]), remain mostly unchanged during the six-month period for all the three conditions tested.

Figure 6 also allows to make some considerations on the vesicles size. In particular, making a rapid digression, it can be seen that the values of number distribution size of about 50–80 nm (Figure 6A) deviate from those of Z-average, which are instead of about 200–300 nm (Figure 6B), indicating the presence of a smaller and larger component in the sample. This could be attributed to the presence of a possible lipid vesicles aggregation, as already stated in Section 3.2.2, or to the eventual release of metal debris from the sonicator tip during the homogenization phase, thus requiring a further centrifugation step to be removed, which has not been performed in this work. This would also be useful for lowering PDI values.

**Figure 7:**
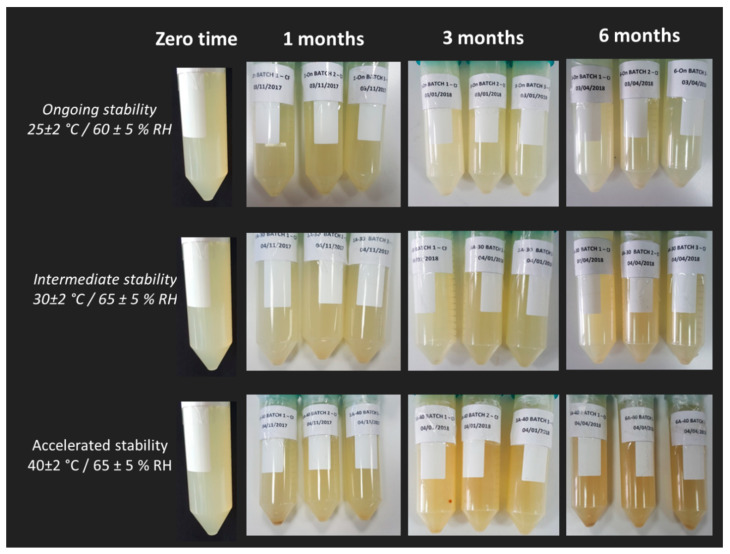
Visual appearance of samples stored at 25 °C ± 2 °C/60% RH ± 5% RH (ongoing stability), 30 °C ± 2 °C/65% RH ± 5% RH (intermediate stability) and 40 °C ± 2 °C/75% RH ± 5% (accelerated stability) at zero time and after 1, 3, and 6 months.

**Figure 8:**
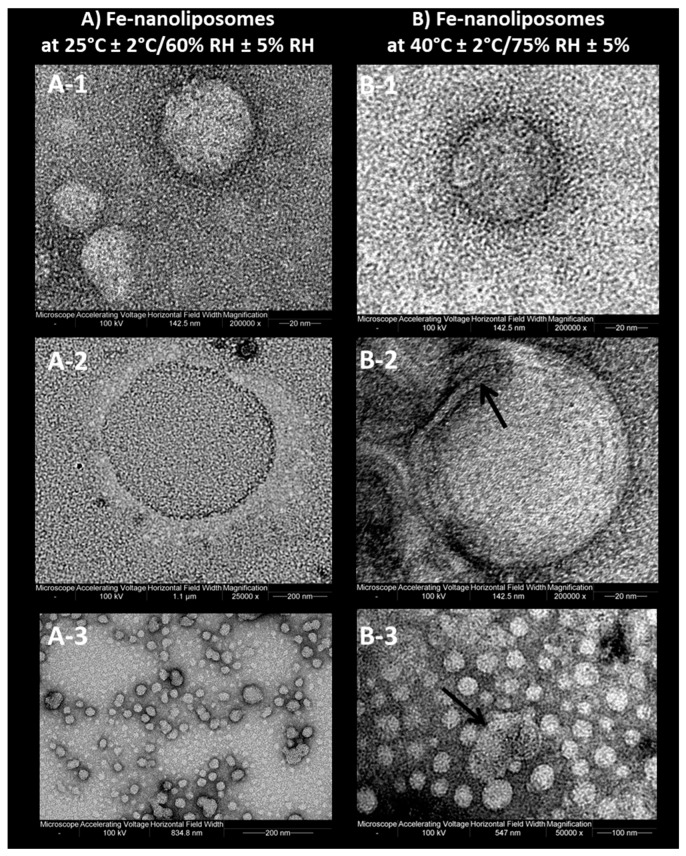
Transmission electron micrographs of (**A**) nanoliposomes stored at 25 °C ± 2 °C/60% RH ± 5% RH (ongoing stability) and (**B**) nanoliposomes stored at 40 °C ± 2 °C/75% RH ± 5% (accelerated stability) after six months.

Going back to the stability assessment, after three months of storage only the Z-average of liposomes at intermediate and accelerated conditions started to slightly decrease, probably due to the nanoliposomes aggregation and the consequent collapse of larger structures which were no longer detectable by Dynamic Light Scattering. This result is in line with the findings of the visual analysis: as shown in Figure 7, while the sample tested for the ongoing stability appears in the form of a perfect suspension even after six months, the sample stored at 40 °C ± 2 °C/75% RH ± 5% presents a yellow color and accumulation of lipid material on the bottom of the test tube after one month. In Figure 8, the differences between the two sample are even more noticeable. In particular, it can be seen that the liposomes structure remains whole after six months, as shown in Figure 8A-1, B-1, A-2, B-2, which are all very similar (the multilayer like structure visible in Figure 8B-2 can be ascribable to a stain and lipids interaction during TEM visualization). Some aggregates can be instead observed for the accelerated stability tested samples (Figure 8B-3), thus confirming the zeta average and the visual analysis results.

Variations of the loading efficiency over time and the impact on the liposomal iron formulation quality attributes has been also evaluated. As shown in Figure 6E, the E.E.% reduction after the first month of storage is only about the 6% for liposomes maintained at 25 °C ± 2 °C/60% RH ± 5% RH and of 9% and 12% for samples stored at 30°C ± 2°C/65% RH ± 5% RH and 40 °C ± 2 °C/7 5% RH ± 5%, respectively. After three months of storage, about 80% of the iron is still encapsulated in nanoliposomes maintained at ambient conditions and more than 70% in nanoliposomes tested for intermediate and accelerated conditions. Finally, after six months, about the 60% of the iron is still encapsulated in nanoliposomes stored at 25 °C ± 2 °C/60% RH ± 5% RH, and at 30 °C ± 2 °C/65% RH ± 5% RH, and about the 50% is still inside the carriers stored at 40 °C ± 2 °C/75% RH ± 5%.

The E.E.% decreases faster for samples tested for accelerated stability, this because the hydrolysis rate of phospholipid increases with an increase in the storage temperature, which results in an increase in the leakage of encapsulated iron, in agreement with what has been shown by Pan et al. for astaxanthin-liposomes [63]. In general, an excellent liposome ability to maintain the iron load even in stress conditions was observed.

Finally, as can be observed in Figure 6F, the percentage change in volume is statistically negligible for all samples at three different conditions for the 6 months of observation. Small volume variations can be noted just for the samples at 40 °C ± 2 °C/75% RH ± 5% due to higher temperatures.

#### 3.2.4. In Vitro Bioavailability Studies

Intracellular and extracellular ferritin, an iron storage protein, was determined in CaCo-2 cells and used as marker of iron absorption for the carrier’s bioavailability evaluation. CaCo-2 cells are a human colon epithelial cancer cell line used as a model of human intestinal absorption of drugs and other compounds [35].

**Figure 9:**
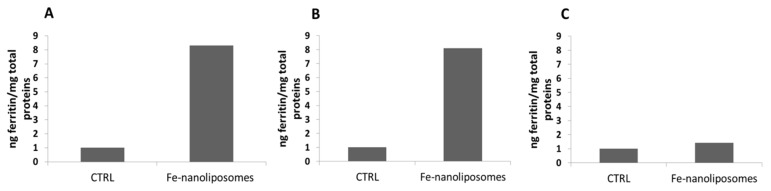
Caco-2 cell iron absorption from nanoliposomes-ferrous sulfate complexes: (**A**) intracellular ferritin levels of not treated cells (CTRL) and after Fe-nanoliposomes treatment; (**B**) intracellular ferritin levels of not treated cells (CTRL) and after the uptake of Fe-nanoliposomes stored for two months at 4 °C; (**C**) Extracellular ferritin levels of not treated cells (CTRL) and after the uptake of Fe-nanoliposomes stored for two months at 4 °C.

The nanoliposomes-iron formulation tested demonstrated a very high iron absorption in Caco-2 cells. As shown in Figure 9A the ferritin levels produced by the cells treated with 20 µM nanoliposomes-iron complexes were 8 times higher compared to those expressed by the not treated cells and the result remains unchanged for cells treated with the same sample stored at 4 °C for two months (Figure 9B). Furthermore, as a guarantee of a good iron absorption, it was shown that 24 h after the treatment with the complexes, the levels of extracellular ferritin, i.e., the protein found in the supernatants, were approximately equal to those of the control sample (not treated cells); thus, the protein was not released in a significant amount into the extracellular Caco-2 environment (Figure 9C). The explanation may lie in the use of the most bioabsorbable form of iron, the ferrous sulfate, and in its encapsulation inside carriers that mimic the composition of the cellular membranes with which they blend promoting the iron transport across the plasma membrane. Moreover, as also highlighted in a work by Zariwala et al. in which ferrous-sulfate-loaded solid lipid nanoparticles were produced for oral iron delivery, due to their bioadhesive properties, lipid carriers can increase the cellular uptake by adhering to the GI tract, internalizing the encapsulated active molecule or changing the barrier function of the GI tract by acting as permeation enhancers [46].

**Figure 10:**
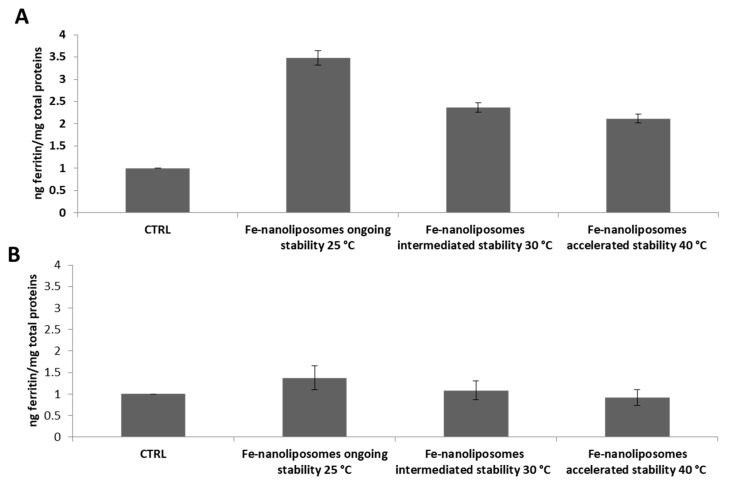
Caco-2 cell iron absorption from nanoliposomes-ferrous sulfate complexes tested for their stability at different conditions for six months. (**A**) Intracellular ferritin levels and (**B**) extracellular ferritin levels of not treated cells (CTRL) compared to the protein amount expressed after the uptake of Fe-nanoliposomes maintained, respectively, at 25 ± 2 °C/60 ± 5% RH at environment condition and at 30 ± 2 °C/65 ± 5% RH and 40 ± 2 °C/65 ± 5% RH at thermostated conditions.

The bioavailability of nanoliposomes–iron complexes was also studied for the samples tested for ongoing, intermediate, and accelerated stability showing an excellent ability of the liposomal structures to keep the ferrous sulfate intact while continuing to guarantee its bioavailability. Therefore, intracellular and extracellular ferritin was determined for cells treated with the sample maintained at 25 ± 2 °C/60 ± 5% RH at environment condition and those stored at 30 ± 2 °C/65 ± 5% RH and at 40 ± 2 °C/65 ± 5% RH at thermostated conditions for six months. As shown in Figure 10A, cells treated with the complexes maintained at 25 ± 2 °C/60 ± 5% RH express 3.4 times more ferritin compared to the not treated cells and those treated with complexes maintained at 30 ± 2 °C/65 ± 5% RH and at 40 ± 2 °C/65 ± 5% RH synthesize 2.3 and two times more ferritin, respectively, compared to the control sample, showing not only the liposomal stability but also the chemical integrity of the contained ferrous sulfate. Finally, the levels of extracellular ferritin 24 h after the treatment with nanoliposomes containing iron tested for stability were approximately equal to those of the not treated cells showing again the good iron absorption by Caco-2 cells (Figure 10B).

### 3.3. A Comparison between the Simil-Microfluidic Apparatus and Two Classical Bench Scale Techniques

The relevant characteristics of iron nanoliposomes produced by the simil-microfluidic method have been compared with those of loaded ferrous sulfate vesicles produced with two more conventional techniques, prepared at the same operating conditions and the 0.01 *w*/*w* Fe/total components formulation. Table 4 summarizes the results of the comparison in terms of number distribution size, PDI, zeta potential, and E.E. Size features are comparable except for a slight reduction for nanoliposomes produced by ultrasound-assisted Thin Film Hydration method and an increase in PDI by using the Ethanol Injection method. Zeta potential values as well as the E.E. remain high and mostly similar. It is possible to assert that the simil-microfluidic method presents advantages, such as being a tunable technique (liposomes dimensional features can change varying the flow conditions), having a potential high productivity (it is possible to work at continuous regime), working at room condition (no high pressure or temperature values are required), and keeping to a minimum the use of solvents.

In particular, the conventional Thin Film Hydration method [64] followed by an ultrasound-assisted size reduction process [38] was used in our previous works for the production of SUVs encapsulating different kind of vitamins [65] and nucleic-acid-based drugs (NABDs) [66,67] for nutraceutical and pharmaceutical applications, respectively. Despite the possibility in achieving liposomes on nanometric scale (about a 49–86 nm distribution range size) due to the versatility of the size reduction process developed, obtaining high loads for the most of the active molecules used, the technique suffers from the impossibility to scale up the process. The volumes in output are small, with long process times. Indeed, if also considering the higher capacity of a standard size, commercially available round bottom flask, the resulting volume of product obtainable in a given time (usually 3-4 h are necessary just to the solvent evaporation step) is small and finite, the situation is not different for the ethanol injection technique that, despite being a more rapid technique, presents a maximum syringe volume of 50 mL. Moreover, the process is discontinuous and not controllable. The semil-microfluidic apparatus developed has not limits in the production; in particular, in just 2.5 min, it is possible to produce 110 mL of nanoliposomes solution. As visible in Table 5, through the use of our apparatus the yield of the process, intended as the ratio of the number of liposomes produced in one batch for unit volume to the time spent for their production, is about 2 × 10^12^ (1/mL h) while with the conventional technique the yield decreases becoming about 9 × 10^9^ (1/mL h) and 5.4 × 10^11^ (1/mL h), respectively, when the Thin Film Hydration and the Ethanol Injection methods are adopted.

### 3.4. Scale Up of the Ultrasound Assisted Homogenization Process

**Figure 11:**
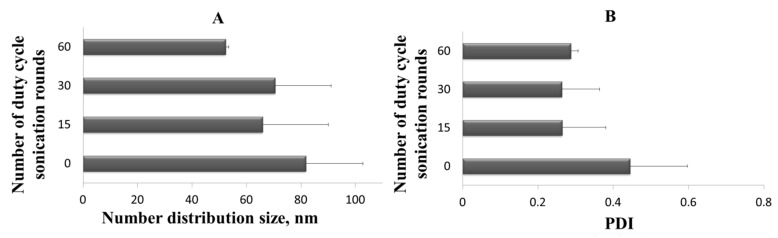
(**A**) Liposomes number distribution size (the mean of the number size distribution) after different number of duty cycle sonication rounds; (**B**) Polydispersity Index (PDI) of liposomes after different number of duty cycle sonication rounds. Results are expressed as average of three determinations and reported along with the standard deviation.

In order to scale up the ultrasound assisted homogenization process, the duty cycle irradiation time must be kept constant and the 6 mm tip with a 100% amplitude, the power to be delivered, and thus the number of sonication cycles able to amplifying the sound wave on the entire batch volume produced (110 mL) generating the same dimensional features founded for sonicated sample aliquots (1 mL) must be used. The effect of different time of sample exposure to ultrasound on the vesicles dimensional features was studied after 0, 15, 30, and 60 rounds of duty cycle sonication, and the results are reported in Figure 11A,B.

As a result of the homogenization process scale-up, it was possible to observe a reduction of PDI (Figure 11B) after only 15 sonication rounds, thus observing values that can be compared with those obtained on a small scale, operating with 1 mL sample aliquot and the 45% amplitude (Section 3.2.2). In conclusion, the homogenization step has proved to be an effective and easily scalable method therefore was included in the ferrous sulfate production process by using the new simil-microfluidic apparatus configuration without changes in iron encapsulation efficiency.

## 4. Conclusions

Ferrous sulfate loaded nanoliposomes with high encapsulation efficiencies (more than 97% E.E.), good dimensional features (about 80 nm, with a narrow size distribution), stable at short and long term conditions (at 4 °C for six days; at 25 °C, 30 °C and 40 °C for six months), and with an excellent bioavailability in Caco-2 cell line, were successfully produced through the use of the built simil-microfluidic apparatus, obtaining an elevated process yield if compared to the classical bench scale techniques. Moreover, massive production of nanoliposomes can be achieved due to the operative modality of the developed apparatus, and a control on particles size and size distribution is obtainable by varying the feed solutions volumetric flow rate ratio or lipids concentration.

Taking into account that “less waste” and “less energy” are the central topics of food manufacturers, the developed apparatus could make iron-nanoliposomal-based nutraceutical products cheaper due to a more efficient and sustainable production process, avoiding the use of toxic solvents and onerous conditions. The simil-microfluidic apparatus developed could have a great impact on the nutraceutical processing industries by drastically reducing process time and costs.

## Figures and Tables

**Table 1:** Reynolds number (R_e_) relative to the polar phase, the organic phase, and the hydroalcholic solution at the different volumetric flow rates ratio tested.

V, mL/min	R_e_ Number
*Polar Phase*	
40.0	169.7
60.0	254.6
80.0	339.5
160	679.0
*Organic Phase*	
4.00	34.9
*Hydroalcholic Phase*	
44.0	311.4
64.0	452.6
84.0	594.5
164.0	1160

**Table 2:** Sonicated and not sonicated liposomes number distribution size (the mean of the number size distribution), polydispersity index (PDI), and Zeta potential produced at different weight ratio of ferrous sulfate to the total formulation components. Results are expressed as average of three determinations and reported along with the standard deviation (SD).

	Unloaded Vesicles	0.06 *w*/*w*	0.035 *w*/*w*	0.02 *w*/*w*	0.01 *w*/*w*
• *Not sonicated samples number distribution size and PDI*					
Size (nm) ± SD	71.810 ± 6.97	127.01 ± 36.6	154.08 ± 15.9	103.64 ± 29.1	135.33 ± 11.2
PDI ± SD	0.41 ± 0.03	0.45 ± 0.04	0.76 ± 0.03	0.69 ± 0.08	0.40 ± 0.03
• *Sonicated samples number distribution size and PDI*					
Size (nm) ± SD	50.52 ± 19.7	47.80 ± 6.46	53.40 ± 19.2	65.16 ± 15.5	76.29 ± 16.4
PDI ± SD	0.22 ± 0.01	0.38 ± 0.01	0.52 ± 0.00	0.63 ± 0.12	0.37 ± 0.03
• *Samples zeta potential*					
Zeta Potential (mV) ± SD	−57.87 ± 1.13	−41.05 ± 0.7	−20 ±1.16	−19±0.55	−35.87 ± 2.05

**Table 3:** Theoretical load, effective load, and encapsulation efficiency (E.E.) of sonicated liposomes produced at different weight ratio of ferrous sulfate to the total formulation components. Results are expressed as average of three determinations and reported along with the standard deviation (SD).

Weight Ratio of Ferrous Sulfate to the Total Formulation Components	0.06 *w*/*w* Fe/Total Components	0.035 *w*/*w* Fe/Total Components	0.02 *w*/*w* Fe/Total Components	0.01 *w*/*w* Fe/Total Components
Theoretical load ± SD (%)	4.23	3.41	2.30	0.980
Effective load ± SD (%)	0.990 ± 0.03	1.28 ± 0.32	1.22 ± 0.20	0.930 ± 0.13
E.E. ± SD (%)	22.33 ± 0.580	42.14 ± 6.74	52.20 ± 1.41	96.63 ± 2.70

**Table 4:** Number distribution size (the mean of the number size distribution), PDI, Zeta Potential and encapsulation efficiency (E.E.) of ferrous sulfate loaded nanoliposomes produced with three different techniques adopting the 0.01 *w*/*w* Fe/total components formulation. Results are expressed as average of three determinations and reported along with the standard deviation (SD).

Production Technique	Simil-Microfluidic Setup	Thin Film Hydration	Ethanol Injection
Number distribution size (nm) ± SD	76.29 ± 16.4	49.85 ± 1.79	74.53 ± 9.81
PDI ± SD	0.37 ± 0.03	0.30 ±0.02	0.52 ± 0.04
Zeta Potential (mV) ± SD	−35.87 ±2.05	−30.20 ±1.27	−37.27 ± 2.26
E.E. ± SD (%)	96.63 ± 2.7	97.98 ± 2.2	100.0 ± 0.00

**Table 5:** Comparison in terms of process yield between the productions of the simil-microfluidic setup and two classical bench scale methods. Volumes of 110 mL (one batch volume), 3000 mL (maximum round bottom flask volume), and 50 mL (maximum syringe volume) were considered respectively for SMF, ultrasound-assisted Thin Film Hydration (TFH) and Ethanol Injection (EI) techniques, with a production time of 2.5 min, 10 min, and 24 h.

Production Technique	Simil-Microfluidic, SMF Setup	Thin Film Hydration, TFH	Ethanol Injection, EI
Maximum volume producible, mL	No limits	3000	50
Yield for a batch, 1/(mL h)	2 × 10^12^	8.98 × 10^9^	5.42 × 10^11^
